# *Lactiplantibacillus plantarum* AR113 Exhibit Accelerated Liver Regeneration by Regulating Gut Microbiota and Plasma Glycerophospholipid

**DOI:** 10.3389/fmicb.2021.800470

**Published:** 2022-01-13

**Authors:** Chunliang Xie, Zhoumei Zhang, Manyi Yang, Cha Cao, Yingjun Zhou, Zuohua Zhu, Wenbing Gong, Chao Xu, Li Yan, Zhenxiu Hu, Lianzhong Ai, Yuande Peng

**Affiliations:** ^1^Institute of Bast Fiber Crops, Chinese Academy of Agricultural Sciences, Changsha, China; ^2^Shanghai Engineering Research Center of Food Microbiology, School of Medical Instrument and Food Engineering, University of Shanghai for Science and Technology, Shanghai, China; ^3^Department of Hepatobiliary and Pancreatic Surgery, NHC Key Laboratory of Nanobiological Technology, Xiangya Hospital, Central South University, Changsha, China

**Keywords:** *Lactiplantibacillus plantarum*, partial hepatectomy, liver regeneration, gut microbiota, plasma metabolites, glycerophospholipid

## Abstract

Emerging evidence indicates that probiotics have been proved to influence liver injury and regeneration. In the present study, the effects of *Lactiplantibacillus plantarum* AR113 on the liver regeneration were investigated in 70% partial hepatectomy (PHx) rats. Sprague-Dawley (SD) rats were gavaged with *L. plantarum* AR113 suspensions (1 × 10^10^ CFU/mL) both before and after partial hepatectomy. The results showed that *L. plantarum* AR113 administration 2 weeks before partial hepatectomy can accelerate liver regeneration by increased hepatocyte proliferation and tumor necrosis factor-α (TNF-α), hepatocyte growth factor (HGF), and transforming growth factor-β (TGF-β) expression. Probiotic administration enriched *Lactobacillus* and *Bacteroides* and depleted *Flavonifractor* and *Acetatifactor* in the gut microbiome. Meanwhile, *L. plantarum* AR113 showed decline of phosphatidylethanolamine (PE), phosphatidylcholine (PC), phosphatidyl serine (PS), and lysophosphatidyl choline (LysoPC) levels in the serum of the rats after the *L. plantarum* AR113 administration. Moreover, *L. plantarum* AR113 treated rats exhibited higher concentrations of L-leucine, L-isoleucine, mevalonic acid, and lower 7-oxo-8-amino-nonanoic acid in plasma than that in PHx. Spearman correlation analysis revealed a significant correlation between changes in gut microbiota composition and glycerophospholipid. These results indicate that *L. plantarum* AR113 is promising for accelerating liver regeneration and provide new insights regarding the correlations among the microbiome, the metabolome, and liver regeneration.

## Introduction

Liver diseases are a major medical problem for health care systems worldwide (Chowdhury et al., 2021). Partial hepatectomy and liver transplantation are currently the only curable methods for patients with hepatocellular carcinoma and cirrhosis (Yagi et al., 2020). However, complications like biliary leakage with consecutive bacterial peritonitis have a severe negative impact on the post-operative course (Tanemura et al., 2018). Therefore, the liver’s remarkable capacity to regenerate after surgery determines the long-term prognosis and quality of life of patients.

Liver regeneration is an orchestrated biological process that includes sequential changes in gene expression, growth factor production, and tissue remodeling (Michalopoulos and Bhushan, 2021). Following liver resection, hepatocytes, which are not terminally differentiated, exhibit substantial proliferative capacity. Many cytokines, notably HGF, epidermal growth factor, transforming growth factor-α (TGF-α), interleukin-6 (IL-6), and TNF-α, which are involved in liver regeneration, have been identified and extensively reviewed (Hoffmann et al., 2020). However, liver regeneration research has typically focused on signaling pathways intrinsic to the liver, overlooking those derived from the gut.

Due to the intestinal-liver axis interaction, there are natural and close links between the gut and the liver in terms of anatomical structure and physiological function. Gut microbiota play an important role in different liver diseases (such as non-alcoholic fatty liver disease, cirrhosis, hepatocellular carcinoma, alcoholic liver disease, etc.) (Trebicka et al., 2021). For example, in decompensated liver cirrhosis, gut microbiota composition is changed due to factors such as liver function decline, decreased bile secretion, and hepatic portal hypertension (Crismale and Friedman, 2020). On the contrary, intestinal mucosal permeability is increased, bacterial overgrowth and translocation of intestinal bacteria leads to endogenous infection, which is a common complication of end-stage cirrhosis (Li et al., 2018). It has been noted that liver regeneration is closely associated with alterations in gut microbiota. In the absence of gut microbiota, the normal regeneration function of the liver was significantly inhibited (Adolph et al., 2018). Gut microbiota may indirectly interfere with liver regeneration after partial hepatectomy by inducing systemic or local inflammatory responses through bacterial or endotoxin translocation (Cornide-Petronio et al., 2020). In addition, gut microbiota affects intestinal signaling and enterohepatic circulation of bile acids (BAs) which have been identified as key metabolic signals during liver regeneration (Liu et al., 2015).

Probiotics supplementation is associated with modulation of the gut microbiota to reduce the inflammation cascade and enhance the immune system associated with liver surgery (Nishida et al., 2018). *Pediococcus pentoseceus*, *Lactococcus raffinolactis*, and *Lactobacillus paracasei 19* inhibited bacterial translocations after liver resection in rats, and induced hepatocyte mitosis which was delayed by colonic anastomosis (Seehofer et al., 2004). Treatment with the Linex containing *Lactobacillus* and *Bifidobacterium* alleviated hepatic injury and restored liver function in chronic liver disease patients (Rodes et al., 2014). Although these selected strains have been shown to prevent bacterial infections following abdominal surgery, thus far, the experience with selected probiotics in patients after PHx is limited.

The present study aimed to evaluate the effects of *L. plantarum* AR113 in liver regeneration. The results showed that *L. plantarum* AR113 intervention 2 weeks prior to partial hepatectomy significantly promoted liver regeneration and reduced mortality in animals. In addition, comprehensive analyses of cytokines, gut microbiome, and serum metabolites composition were performed to explore the mechanism underlying the beneficial effects of *L. plantarum* AR113 during the late phases of liver regeneration. In general, our data suggest that *L. plantarum* AR113 administration before PHx may be a promising strategy to accelerate liver regeneration.

## Materials and Methods

### Bacterial Cultures and Growth Conditions

*Lactiplantibacillus plantarum* AR113 was obtained from the Shanghai Engineering Research Center of Food Microbiology, University of Shanghai for Science and Technology (Shanghai, China), which was kept at the China General Microbiological Culture Collection Center, preservation number, CGMCC No. 13909). *L. plantarum* AR113 was stored in 30% glycerol tubes at −80°C. The bacteria were first streaked on Man-Rogosa-Sharpe (MRS) agar plates and cultured in an anaerobic station at 37°C. After 3 days of culture, single colonies of bacteria were activated in MRS liquid medium for 2 generations and cultured at 37°C for 16 h. The bacteria were centrifuged (8,000 × *g*, incubated at 4°C for 10 min) and resuspended with sterile phosphate buffer saline (PBS, pH 7.4) until the final concentration was 1*10^10^ CFU/mL.

### Animals and Partial Hepatectomy

The animal protocol was reviewed and approved by the Animal Care Committee of Institute of Bast Fiber Crops, Chinese Academy of Agricultural Sciences (no. 2020-016). SD rats were housed in steel microisolator cages at 22°C with a 12-h light/dark cycle. Food and water were provided *ad libitum* throughout study. A total of 120 male rats were randomly divided into five groups: (A) control, (B) sham hepatectomy, the sham hepatectomy consisted of laparotomy and mobilization of the liver, (C) PHx, 70% liver resection procedures were performed, (D) AR113+PHx, *L. plantarum* AR113 was given by gastric gavage, which was started 14 days before partial hepatectomy, and continued until 3 or 7 days after the PHx. (E) PHx+AR113, *L. plantarum* AR113 was given by gastric gavage, which was started at partial hepatectomy, and continued until 3 or 7 days after the operation. For rats in groups (C–E), 70% liver resection procedures were performed according to the method published by Higgins and Anderson (Higgins and Anderson, 1931). *L. plantarum* AR113 (suspended in physiological saline) was given to rat by oral gavage at a dose of 10^10^ CFU/mL. Rats in Groups A and B were gavaged the same volume of physiological saline. Rats were killed 3 or 7 days after 2/3 PHx surgery covering the time when hepatocytes are actively proliferating. At the end of the experiment, animals were sacrificed, and liver, blood, and fecal samples were collected.

### Hepatic Regeneration Rate Measurement

The liver regeneration rate was calculated as remnant liver weight/estimated whole liver weight, which was calculated as follows:


Liver⁢Regeneration⁢Rate

=[Wc-(Wa-Wb)]/[Wa-Wb]×100

Wa=Wb/70%

Where Wa is the initial weight of rat liver at the start of PHx, and Wb and Wc are the actual weights of the surgically excised liver tissue and the residual liver tissue at the time points of 3 and 7 days after reperfusion.

### Liver Histology

After rats were sacrificed, a portion of each excised liver was formalin-fixed and sliced into 5 μm thick sections. The sections were then stained with Hematoxylin-Eosin (H&E) for morphological examination. Three H&E-stained levels/sections were examined per specimen. Images were then taken at 20× magnification.

Immunohistochemistry was carried out for Ki-67 to estimate liver proliferation. The sections were incubated with rabbit anti mouse Ki-67 (1:100 Abcam, Cambridge, United Kingdom) as primary antibodies overnight at 4°C. After washing, the sections were incubated with second antibodies HRP polymer detection kit. Digital images were taken around the central vein by using an AxioM1 light microscope (Carl Zeiss, Germany). The number of Ki-67-positive hepatocytes was manually counted in 20 random visual fields at 200X magnification.

### Liver Biochemistry

The concentrations of liver plasma total bilirubin-V (TBil-V), alanine aminotransferase (ALT), aspartate aminotransferase (AST), IL-6, albumin II (ALB II), globulin II (Glo II), total protein (TP), TNF-α, TGF-β, and HGF were determined by using commercial kits as described in the references (Cassano and Dufour, 2019).

### Analysis of the Gut Microbiota

Fecal DNA was manually extracted using QIAamp DNA Stool Mini Kit (Qiagen, Germany). The extracted DNA from each sample was used as the template to amplify the V3 and V4 hypervariable regions of ribosomal 16S rRNA genes on a 454-Junior Genome Sequencer (Roche 454 Life Sciences, Branford, CT, United States) as described. The 16S rRNA genes were amplified by the universal primers F (5′-ACTCCTACGGGAGGCAGCAG-3′) and R (5′-GGACTACHVGGGTWT-CTAAT-3′). The PCR products were purified with AMPure XP beads (Agencourt, Beckman Coulter, Brea, CA, United States) and were sequenced on an Illumina MiSeq platform (Illumina, San Diego, CA, United States). The clean data were clustered into operational taxonomic units (OTUs) with a 97% threshold by Vsearch software (v2.3.4, Vsearch). OTUs were annotated with RDP classifier as described to the Ribosomal Database Project (RDP, database v.11.3). The Chao1 index, Shannon index, and principal coordinates analysis (PCoA) were calculated by QIIME software (version 1.8.0). Linear discriminant analysis effect size (LEfSe) analysis was performed on the Galaxy web platform to identify discriminant taxa among groups. Linear discriminant analysis (LDA) score was used to estimate the effect size of different taxon. Results with LDA score greater than 3.5 were defined as discriminative taxa.

### Plasma Metabolites Analysis

Each 200 μL serum was added to 600 μL pre-cooled methanol: acetonitrile (2:1 = v:v) and vortexed for 1 min. After centrifugation at 2 × 10^4^ *g* for 20 min, supernatant was transferred to a new tube and freeze-dried. The dried samples were re-constituted with 10% aqueous methanol, filtered through 0.22 μm polyvinylidene fluoride membrane, and used for subsequent LC-MS/MS analysis.

To identify the metabolites from plasma, samples were analyzed by LC-MS/MS analysis, which was described in our previous study (Heinrich et al., 1988). The extracts were analyzed by ACQUITY UHPLC system (Waters) coupled to a Xevo G2-XS Q-TOF mass spectrometer, operating in both positive and negative ionization mode. The sample was loaded onto an ACQUITY UPLC BEH C18 (100 mm × 2.1 mm, 1.7 μm) column held at 45°C. The mobile phase consisted of 0.1% (v/v) formic acid (solution A) and acetonitrile contained 0.1% formic acid (solution B), with a flow rate of 0.4 mL/min. The elution profile was set as following: 0 min, 1% B; 1 min, 5% B; 2 min, 30% B; 3.5 min, 60% B; 7.5 min, 90% B; 9.5 min, 100% B; 12.5 min, 100% B; and 12.7 min, 1% B; 16 min, 1%, flow rate, 0.40 mL/min.

The ion source condition settings were as follows: desolvation temperature set at 350°C; capillary voltage set at 30 V; mass spectrometry data range was from 100 to 1,200 m/z. The raw data from the LC-MS/MS were analyzed using the progenesis QI software (Waters Corporation, Milford, United States). The internal standard was used for data QC to test reproducibility of analysis methods. Principle component analysis (PCA) and orthogonal partial least-squares-discriminant analysis (OPLS-DA) were performed using SIMCA-P+12.0.1.0 chemometrics software to visualize the metabolites alterations among the samples. The statistical criteria for preliminary selection of characteristic metabolites were threshold of variable importance in the projection (VIP) from the OPLS-DA greater than 1.0 and *q*-value < 0.05 in a *t*-test. Enriched metabolic pathways were performed using MetaboAnalyst^1^ based on the pathway library from Kyoto Encyclopedia of Genes and Genomes (KEGG).

### Statistical Analysis

Student’s *t*-test was used for comparisons of metabolite levels using the statistical computer package GraphPad Prism version 6 (GraphPad Software Inc., San Diego, CA, United States). Results in the present study were shown as means ± SEM. Statistical comparisons were made using two-way analysis of variance (ANOVA) with Tukey’s *post hoc* test. *P*-values < 0.05 were considered as statistical significance. Columns with different letters differ significantly.

## Results

### *L. plantarum* AR113 Administration Increased Hepatocyte Proliferation and Accelerated Liver Regeneration of 70% Partial Hepatectomy Rats

In order to investigate the effect of *L. plantarum* AR113 administration on liver regeneration, hepatocyte proliferation and hepatic regeneration rate were analyzed. The proliferation of hepatocytes at different groups after two-thirds PHx was evaluated in rats by Ki67 staining. Remarkably, pretreatment with *L. plantarum* AR113 before PHx significantly increased the cell proliferation rate compared with the PHx group at 3 days after PHx (Supplementary Table 1). Because the proliferation of liver cells occurs within 3 days after surgery, there was no significant difference in cell proliferation rates between the PHx group and *L. plantarum* AR113 pretreatment group at 7 days after PHx (Supplementary Table 1). Therefore, *in vivo* proliferation analyses demonstrated that proliferation of hepatocytes was enhanced in the presence of *L. plantarum* AR113 administration.

Rats were sacrificed 3 and 7 days after PHx and their livers were collected and analyzed. Intriguingly, Table 1 shows that *L. plantarum* AR113 pretreatment can significantly reduce rat mortality during PHx. In addition, we also found that hepatic regeneration rate in *L. plantarum* AR113 pretreatment rats was significantly accelerated compared to PHx rats at 7 days after PHx, but there was no significant difference in the hepatic regeneration rate between these two groups at 3 days after PHx, suggesting that it takes a long time for probiotics to promote liver regeneration.

**TABLE 1:** The hepatic regeneration rate and rat mortality after PHx.

	Hepatic regeneration rate		Rat mortality (%)
	3 days after PHx	7 days after PHx	
PHx	45.5% ± 0.57^a^	58.2% ± 0.83^b^	40%
AR113+PHx	46.49% ± 1.09^a^	67.72% ± 1.37^a^	25%
PHx+AR113	40.32% ± 0.65^b^	62.51% ± 0.98^a^	35%

*Different letters indicate significant differences, P < 0.05 (ANOVA followed by Tukey’s HSD test).*

### Effect of *L. plantarum* AR113 Administration on Liver Histological Changes

**FIGURE 1:**
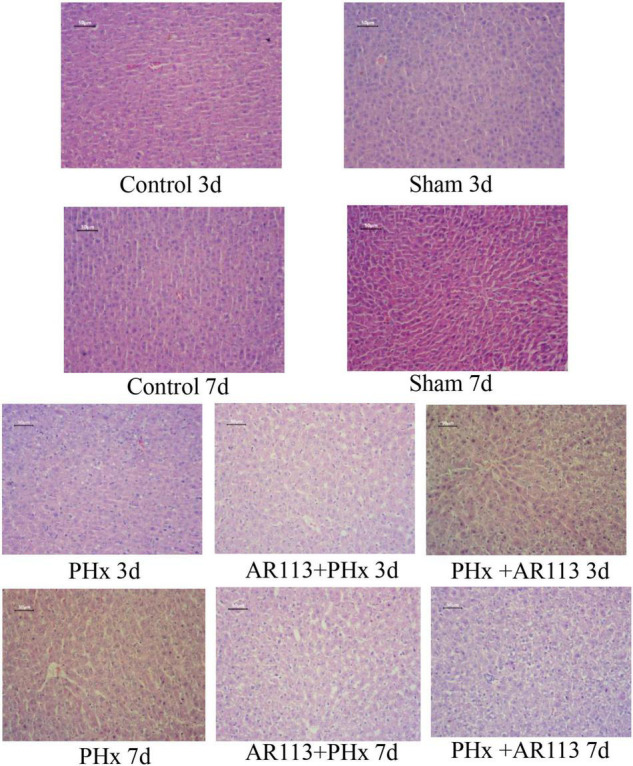
H&E staining 3 and 7 days after PHx of the liver tissue sections of Control, Sham, PHx, AR113+PHx, and PHx+AR113 groups. Scale bar = 50 μm for H&E.

The liver H&E staining results of the rats in all groups are presented in Figure 1. In the Control and Sham groups, the liver showed a normal structure with well-preserved cell morphology and a prominent nucleus. After PHx, the liver structure showed crypt structure atrophy, mucosal epithelium impairment, and decreased goblet cells. However, the histological changes were reversed by *L. plantarum* AR113 administrations, evidenced by a loss of swollen hepatocytes, cytoplasmic vacuolization, and fat vacuoles.

### Effect of *L. plantarum* AR113 Administration on Liver Function, Cytokines, and Growth Factors

In order to investigate the effect of *L. plantarum* AR113 administration on liver function, the contention of ALT, AST, TP, ALB II, TBil-V, and Glo II were detected. Compared with Control and Sham groups, the ALT, AST, and TBil-V levels in PHx rats were significantly increased and ALB II, Glo II, and TP were significantly decreased. Serum ALT, AST, and TBil-V levels were rapidly elevated at Day 3 after PHx (Supplementary Figure 1), and declined at Day 7 after PHx (Supplementary Figure 2). In contrast, serum ALB II, Glo II, and TP were decreased sharply at Day 3 after PHx (Supplementary Figure 1), and could increase at Day 7 after PHx (Supplementary Figure 2). *L. plantarum* AR113 administration had no significant effect on liver function (Supplementary Figure 2).

**FIGURE 2:**
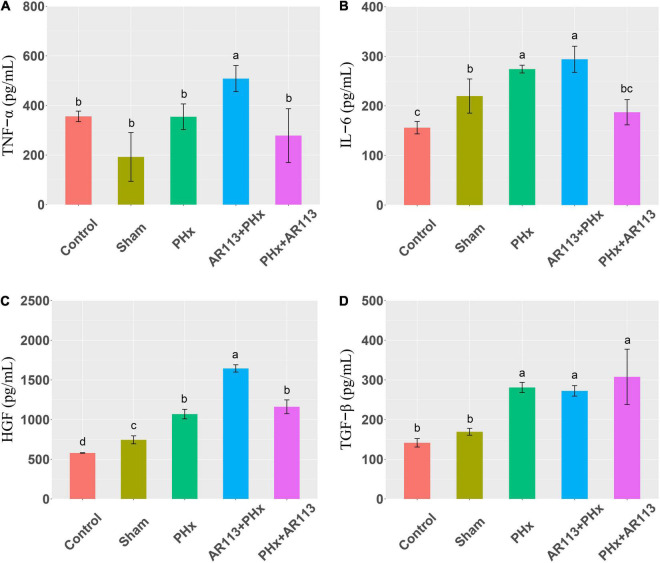
Cytokines and growth factors at 3 days after PHx among the Control, Sham, PHx, AR113+PHx, and PHx+AR113 groups. **(A)** Changes in TNF-α levels; **(B)** Changes in IL-6 levels; **(C)** Changes in HGF levels; **(D)** Changes in HGF-β levels. Different letters indicate significant differences, *P* < 0.05 (ANOVA followed by Tukey’s HSD test).

**FIGURE 3:**
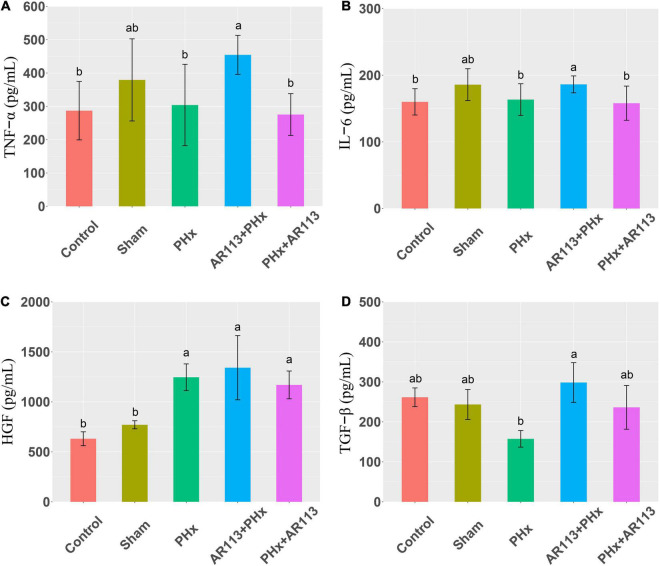
Cytokines and growth factors at 7 days after PHx among the Control, Sham, PHx, AR113+PHx, and PHx+AR113 groups. **(A)** Changes in TNF-α levels; **(B)** Changes in IL-6 levels; **(C)** Changes in HGF levels; **(D)** Changes in HGF-β levels. Different letters indicate significant differences, *P* < 0.05 (ANOVA followed by Tukey’s HSD test).

Cytokines and growth factors have prominent roles in liver regeneration. Serum cytokine and growth factors at 3 days after PHx levels are shown in Figure 2. Compared with the PHx group at 3 days after PHx, TNF-α and HGF were significantly increased by AR113 pretreatment, while TGF-β and IL-6 did not change significantly. At 7 days after PHx, TNF-α, IL-6 and TGF-β were significantly increased by AR113 pretreatment compared with PHx group (Figure 3). Interestingly, there was no significant difference in the expression levels of TNF-α, HGF and TGF-β between the PHx group and the PHx+AR113 group.

### Effect of *L. plantarum* AR113 Administration on Microbial Communities

To elucidate the effects of *L. plantarum* AR113 administration on microbial communities, analysis of the 16S rRNA gene sequences of Control, Sham, PHx, AR113+PHx, and PHx+AR113 groups at Day 3 and Day 7 after PHx was conducted. Sequencing of 16S bacterial RNA retrieved an overall number of 38,155∼41,957 reads, 26,774∼37,361 after filtering, which were clustered in 1,329 operational taxonomic units (OTUs).

Alpha diversity (Shannon and Simpson index) analysis showed that at Day 3 after PHx, the Shannon index of the Control group was significantly higher than that of the Sham, PHx, and AR113+PHx groups (Supplementary Figure 3), but had no significant difference as compared with PHx+AR113. However, there was no significant difference in alpha diversity between the Sham, PHx, AR113+PHx, and PHx+AR113 groups. At Day 7 after PHx, there were no significant differences in alpha diversity between groups, suggesting that the reduced diversity of microbial communities associated with PHx had returned to normal levels (Supplementary Figure 4).

**FIGURE 4:**
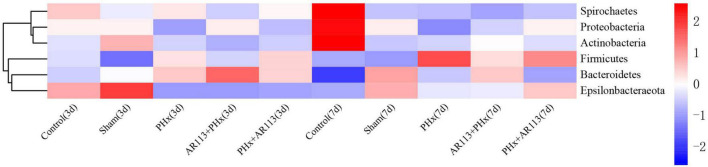
Distribution of relative abundance at the phylum level among Control, Sham, PHx3, AR113+PHx3, and PHx3+ AR113 groups.

**FIGURE 5:**
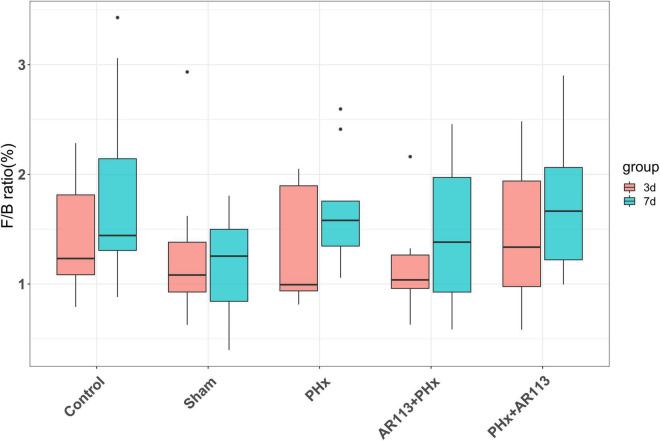
The Firmicutes-to-Bacteroidetes ratio (F/B) of Control, Sham, PHx, AR113+PHx, and PHx+AR113 groups.

Remarkable changes in the microbiota community structure were induced by PHx intervention. The microbes in the Control and Sham groups were more closely clustered relative to PHx and AR113+PHx groups, which is an indication that PHx surgery induced similar microbial composition changes. Distinct changes in microbiota composition have revealed a clear separation between no PHx groups (Control and Sham) and PHx groups (PHx, AR113+PHx, and PHx+AR113) after PHx at Days 3 and 7 (Supplementary Figure 5). *L. plantarum* AR113 treatment induced significant changes in the gut microbial community. At the phylum level, Firmicutes and Bacteroidetes are two major phyla of the domain bacteria in gut microbiota (Figure 4). The abundances of Firmicutes and Bacteroidetes were increased in PHx groups (PHx, AR113+PHx, and PHx+AR113 groups). The Firmicutes-to-Bacteroidetes ratio was calculated, and the results showed that the F/B ratio was elevated in the PHx and PHx+AR113 as compared with the AR113+PHx groups (Figure 5). Proteobacteria abundance in AR113+ PHx and Sham groups was not significantly different but decreased in both PHx and PHx+AR113 groups.

**FIGURE 6:**
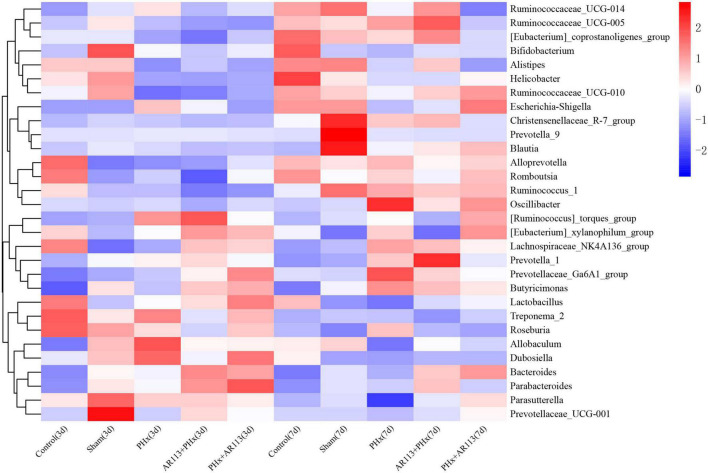
Distribution of relative abundance at the genus level among Control, Sham, PHx3, AR113+PHx3, and PHx3+ AR113 groups.

At the genus level, the abundance of *Lactobacillus* and *Bacteroides* from *L. plantarum* AR113 administration groups was higher than that of PHx and Sham groups (Figure 6). The abundance of *Lachnospiraceae_NK4A136_group* was increased after PHx, which may be related to liver resection. In each genus of Bacteroidetes, the abundance of *Prevotellaceae_Ga6A1_group* has the highest abundance in the AR113+PHx group. In PHx groups (PHx, AR113+PHx, and PHx+AR113), the abundance of the *Prevotella_9* genus was significantly higher than in the Sham group, while the abundance of *Helicobacter* of Proteobacteria in the Sham group was significantly higher than that in the other three groups.

**FIGURE 7:**
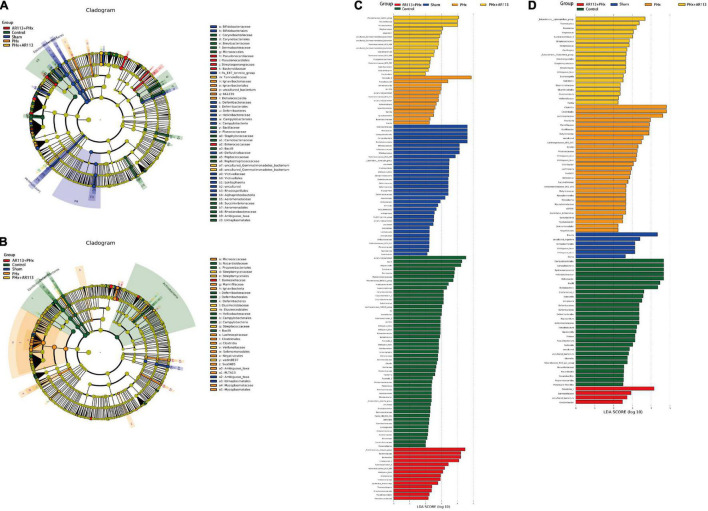
LEfSe analysis among the Control, Sham, PHx, AR113+PHx, and PHx+AR113 groups. Cladogram displays the taxonomic tree of differentially abundant taxa. Histograms represent the LDA scores of bacteria with significant differential abundance between the compared groups, identified by different colors. Features with LDA score ≥ 3. **(A,C)**, 3 days after PHx. **(B,D)**, 7 days after PHx.

We used the LEfSe analysis to identify the specific bacteria phylotypes that were differentially altered among the five groups, the LEfSe algorithm with a logarithmic LDA score cutoff ≥3.0 was then performed (Figure 7 and Supplementary Tables 2, 3). Three days after PHx, the most differentially abundant gut microbiota in group PHx were *Prevotella_9*, *Faecalibaculum*, *Dehalococcoidia*, and *661239*. The gut microbiota enriched in the AR113 administration group (AR113+PHx and PHx+AR113) were *Ruminococcus_torques_group*, *Bacteroidaceae*, *Bacteroides*, *Coprococcus_2*, *Ruminiclostridium_9*, *Ruminococcaceae_ UCG_004*, *Ambiguous_taxa*, *Prevotellaceae_Ga6A1_group*, *Tannerellaceae*, *Parabacteroides*, *Mogibacterium*, and *Atopobium*. Seven days after PHx, the most differentially abundant gut microbiota in group PHx were *Clostridia*, *Clostridiales*, *Lachnospiraceae*, *Roseburia*, *Marinifilaceae*, *Oscillibacter, Butyricimonas, uncultured, Lachnospiraceae_UGG_001*, *PLTA13*, *Micrococcaceae*, *Ambiguous_taxa*, and *Odoribacter*. The gut microbiota enriched in the *L. plantarum* AR113 administration group were *Prevotella_1*, *Eubacterium_xylanophilum_group*, *Thermotunica*, *Roseiarcus*, *Vagococcus*, and *Ruminiclostridium_5*. These results suggest that administration of probiotics can significantly alter the composition of gut microbiota after PHx.

### Effect of *L. plantarum* AR113 Administration on Overall Plasma Metabolite Content

The metabolic profiles were acquired using LC–MS/MS, and 4297 metabolites were identified. Then, multivariate analysis was conducted after data normalization. OPLS-DA model between the PHx and AR113+PHx groups at Day 3 and Day 7 was established and differentially abundant metabolites were derived from this model with a VIP > 1 and a *P*-value < 0.05. There were 68 and 74 differentially expressed metabolites identified between the PHx and AR113+PHx groups at Day 3 and Day 7, respectively. Ultimately, 17 metabolites differentially expressed both in Day 3 and Day 7 were selected (Table 2). Of these metabolites, L-isoleucine, L-leucine, 3-O-Methylniveusin A, piperidine, PA(22:0/a-25:0), PI(20:3(5Z,8Z,11Z)/20:3(8Z,11Z,14Z), and mevalonic acid showed an increase in the AR113+PHx group compared with the PHx group. 1-Arachidonoylglycerophosphoinositol, palmitic amide, 1-(6-[3]-ladderane-hexanoyl)-2-(8-[3]-ladderane-octanyl)-sn-glycerophosphoethanolamine, 7-oxo-8-amino-nonanoic acid, and 5S-HETE di-endoperoxide showed a decrease in the PHx group.

**TABLE 2:** Summary of the differentially expressed metabolites between PHx and AR113+PHx groups.

m/z	Ion mode	Metabolites	VIP	*P*-value	FC
132.10171	Pos	L-Isoleucine	3.10	3.0952	0.77
203.08349	Neg	Phenylalanyl-Glycine	3.95	0.0218	0.80
130.08765	Neg	L-Leucine	1.99	0.0017	0.73
881.19349	Pos	[Gallocatechin(4alpha->8)]2catechin	1.50	0.0009	0.44
881.44495	Pos	1-[2,4-dihydroxy-5-(3-methylbut-2-en-1-yl)phenyl]-2-hydroxy-3-[4-hydroxy-3-methoxy-5-(3-methylbut-2-en-1-yl)phenyl]propan-1-one	1.70	0.0006	0.46
403.23215	Pos	5S-HETE di-endoperoxide	2.30	0.0000	32.63
186.11375	Neg	7-oxo-8-amino-nonanoic acid	2.57	0.0004	2.81
129.05583	Neg	Mevalonic acid	1.35	0.0255	0.69
256.26275	Pos	Palmitic amide	4.28	0.0264	1.69
881.6963	Pos	PA(22:0/a-25:0)	1.61	0.0005	0.46
763.53652	Pos	1-(6-[3]-ladderane-hexanoyl)-2-(8-[3]-ladderane-octanyl)-sn-glycerophosphoethanolamine	1.75	0.0001	1.86
619.28723	Neg	1-Arachidonoylglycerophosphoinositol	1.94	0.0005	1.53
909.54695	Neg	PI(20:3(5Z,8Z,11Z)/20:3(8Z,11Z,14Z))	1.98	0.0185	0.68
254.17573	Neg	Labienoxime	1.29	0.0000	0.34
407.17245	Neg	3-O-Methylniveusin A	2.58	0.0176	0.70
146.05992	Pos	4-formyl Indole	1.39	0.0074	0.82
86.096214	Pos	Piperidine	2.73	0.0054	0.77

*Pos, positive ion mode; Neg, negative ion mode.*

We also compared the plasma metabolite changes between the PHx and PHx+AR113 groups. There were 135 and 115 differentially expressed metabolites identified between the PHx and PHx+AR113 groups at Day 3 and Day 7, respectively. There were 26 metabolites differentially expressed both in Day 3 and Day 7 (Table 3). The probiotic group showed greater reductions in 4-phosphopantothenoylcysteine, chrycorin, PI(20:3(5Z,8Z,11Z)/20:3(8Z,11Z,14Z)), PI(20:4(5Z, 8Z,11Z,14Z)/18:0), PC(22:4(7Z,10Z,13Z,16Z)/22:6(4Z,7Z,10Z, 13Z,16Z,19Z)), PC(22:6(4Z,7Z,10Z,13Z,16Z,19Z)/22:4(7Z,10Z, 13Z,16Z)), 1-Oleoylglycerophosphoserine, and 1-(2-methoxy- 6Z-heptadecenyl)-sn-glycero-3-phosphoserine. In contrast, PE(P-16:0/0:0), sphinganine 1-phosphate, mevalonic acid, PE(18:1(9Z)/0:0), LysoPE(18:2(9Z,12Z)/0:0), LysoPE(20:4(5Z, 8Z,11Z,14Z)/0:0), LysoPE(0:0/20:4(5Z,8Z,11Z,14Z)), L-leucine, and L-isoleucine were upregulated in the PHx+AR113 group compared with the PHx group.

**TABLE 3:** Summary of the differentially expressed metabolites between PHx and PHx+ AR113 groups.

m/z	Ion mode	Metabolites	VIP	*P*-value	FC
132.10171	Pos	L-Isoleucine	3.38	0.0014	0.75
130.08765	Neg	L-Leucine	2.28	0.0083	0.70
552.27106	Neg	Vignatic acid A	3.58	0.0000	3.18
355.06781	Pos	6-[5-(2-carboxyeth-1-en-1-yl)-2,3-dihydroxyphenoxy]-3,4,5-trihydroxyoxane-2-carboxylic acid	3.87	0.0000	1.79
403.23215	Pos	5S-HETE di-endoperoxide	2.28	0.0000	21.29
186.11375	Neg	7-oxo-8-amino-nonanoic acid	2.85	0.0003	3.31
129.05583	Neg	Mevalonic acid	1.87	0.0035	0.56
506.32298	Neg	PC(17:1(10Z)/0:0)	1.33	0.0280	0.89
904.58372	Pos	PC(22:6(4Z,7Z,10Z,13Z,16Z,19Z)/22:4(7Z,10Z,13Z,16Z))	3.28	0.0004	1.99
904.5835	Pos	PC(22:4(7Z,10Z,13Z,16Z)/22:6(4Z,7Z,10Z,13Z,16Z,19Z))	1.83	0.0000	1.94
478.29196	Pos	LysoPE(18:2(9Z,12Z)/0:0)	2.93	0.0433	0.67
500.277	Neg	LysoPE(20:4(5Z,8Z,11Z,14Z)/0:0)	3.30	0.0276	0.69
438.29619	Pos	PE(P-16:0/0:0)	2.83	0.0200	0.51
500.27645	Neg	LysoPE(0:0/20:4(5Z,8Z,11Z,14Z))	1.48	0.0211	0.71
763.53652	Pos	1-(6-[3]-ladderane-hexanoyl)-2-(8-[3]-ladderane-octanyl)-sn-glycerophosphoethanolamine	1.20	0.0096	1.47
885.54815	Neg	PI(20:4(5Z,8Z,11Z,14Z)/18:0)	7.30	0.0163	1.63
909.54695	Neg	PI(20:3(5Z,8Z,11Z)/20:3(8Z,11Z,14Z))	1.29	0.0389	1.46
504.27176	Neg	1-Oleoylglycerophosphoserine	4.20	0.0000	2.42
506.28682	Neg	1-(2-methoxy-6Z-heptadecenyl)-sn-glycero-3-phosphoserine	3.08	0.0000	2.73
401.07701	Neg	4-Phosphopantothenoylcysteine	2.24	0.0463	1.39
380.25588	Neg	Sphinganine 1-phosphate	2.53	0.0277	0.55
466.33424	Pos	1alpha,25-dihydroxy-24a-homo-22-thiavitamin D3/1alpha,25-dihydroxy-24a-homo-22-thiacholecalciferol	2.23	0.0200	0.61
480.30672	Pos	PE(18:1(9Z)/0:0)	3.11	0.0143	0.61
72.080461	Pos	Pyrrolidine	2.24	0.0008	0.59
86.096214	Pos	Piperidine	3.04	0.0006	0.74
201.03839	Neg	Chrycorin	3.05	0.0007	1.42

*Pos, positive ion mode; Neg, negative ion mode.*

### Effect of *L. plantarum* AR113 Administration on Metabolic Pathways

**FIGURE 8:**
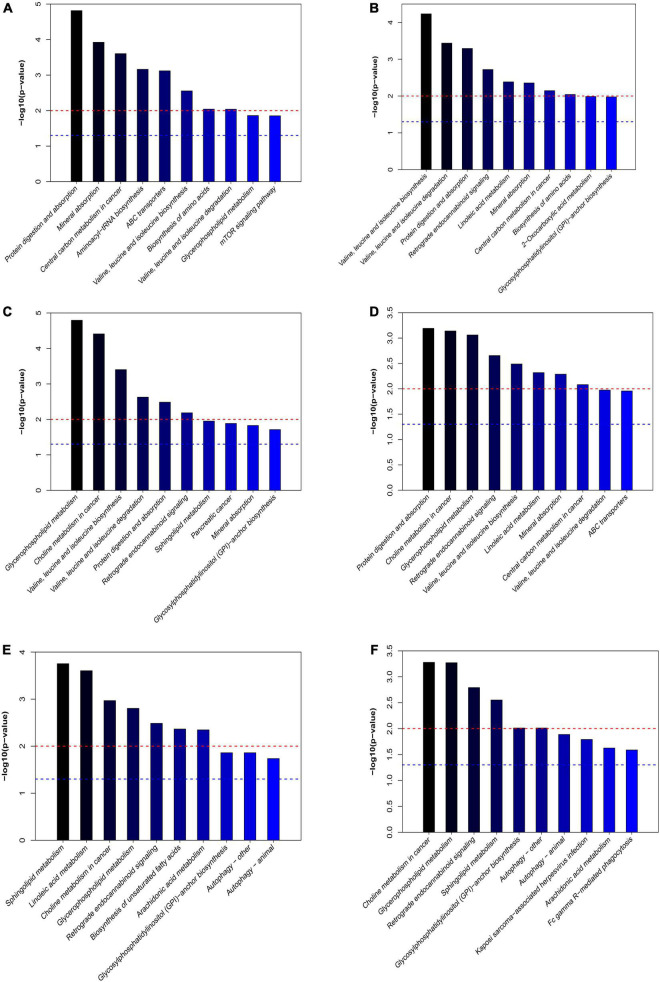
The differentially expressed metabolic pathways among the PHx, AR113+PHx, PHx+AR113 groups of 3 or 7 days after PHx. **(A)** PHx and AR113+PHx groups of 3 days after PHx; **(B)** PHx and AR113+PHx groups of 7 days after PHx; **(C)** PHx and PHx+AR113 groups of 3 days after PHx; **(D)** PHx and PHx+AR113 groups of 7 days after PHx; **(E)** AR113+PHx and PHx+AR113 groups of 3 days after PHx; **(F)** AR113+PHx and PHx+AR113 groups of 7 days after PHx.

The differential expressed metabolites between PHx and AR113+PHx groups or PHx and PHx+AR113 groups were mapped to the KEGG database^2^ for metabolic pathway construction (Figure 8). Intriguingly, the differentially expressed metabolites between PHx and AR113+PHx groups were mainly associated with protein digestion and absorption, choline metabolism in cancer, glycerophospholipid metabolism, leucine (Leu) and isoleucine (Ile) biosynthesis, valine (Val), Leu and leucine degradation, and mineral absorption pathways. Besides, Val, Leu, and Ile biosynthesis, Val, Leu, and Ile degradation, protein digestion and absorption, mineral absorption, central carbon metabolism in cancer, and biosynthesis of amino acids were differentially expressed between PHx and PHx+AR113 groups. These results indicated that *L. plantarum* AR113 administration accelerated liver regeneration accompanied by a series of changes in metabolism, especially Leu and Ile biosynthesis, Val, Leu, and Ile degradation, and mineral absorption pathways.

In addition, the differentially expressed metabolites pathways between pre-probiotic (AR113+PHx) and post-probiotic (PHx+AR113) treatment groups were also discussed. It was found that different ways of probiotics administration can affect choline metabolism in cancer, glycerophospholipid metabolism, retrograde endocannabinoid signaling, sphingolipid metabolism, glycosylphosphatidylinositol (GPI)-anchor biosynthesis, autophagy, and arachidonic acid (ARA) metabolism pathways.

### The Correlation Between Gut Microbiome and Plasma Metabolome

**FIGURE 9:**
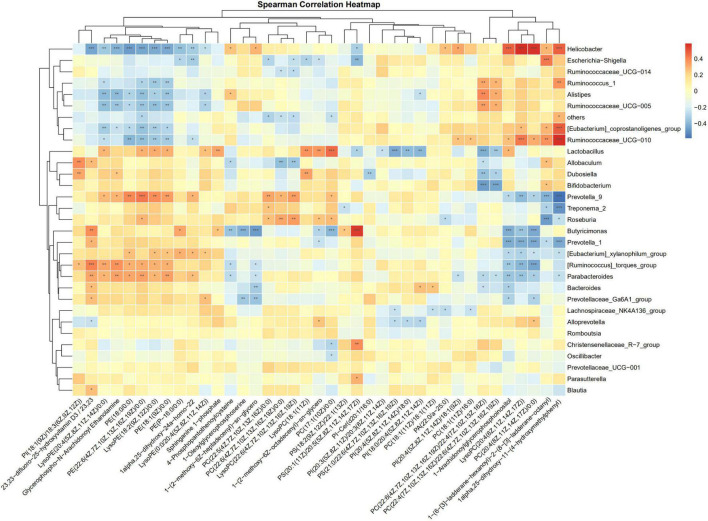
Spearman correlation analysis heatmaps of gut microbiome and glycerolipids. *, **, *** represents a significant difference, *p < 0.05, **p < 0.01, ***p < 0.001 as determined by *t*-test.

The correlations of the discriminative gut microbiome and differential plasma metabolites from Control, Sham, PHx, AR113+PHx, and PHx+AR113 were determined using Spearman’s rank correlation analysis (Figure 9). Taking the correlation analysis of glycerolipids and gut microbiome as an example, the results showed that the relative abundances of *Helicobacter*, *Ruminococcus*, and *Ruminococcaceae* were negatively correlated with serum levels of LysoPE and PE in serum. In contrast, the abundance of *Lactobacillus*, *Allobaculum*, *Dubosiella*, *Prevotella*, *Roseburia*, *Butyricimonas*, *Parabacteroides*, and *Bacteroides* was positively correlated with the circulating levels of LysoPE and PE. In addition, we found PC(18:1(11Z)/18:1(11Z)), PC(22:6(4Z,7Z,10Z,13Z,16Z,19Z)/22:4(7Z,10Z,13Z,16Z), PC(22:4(7Z,10Z,13Z,16Z)/22:6(4Z,7Z,10Z,13Z,16Z,19Z)), LysoPC(20:4(8Z,11Z,14Z,17Z)), and PC(20:4(8Z,11Z,14Z,17Z)/ 0:0) exhibited positive correlations with *Helicobacter*, *Ruminococcus*, *Alistipes*, and *Ruminococcaceae* and negative correlations with *Lactobacillus*, *Allobaculum*, *Dubosiella*, *Bifidobacterium*, *Prevotella_9*, *Butyricimonas*, *Prevotella_1*, *[Eubacterium]_xylanophilum_group, [Ruminococcus]_torques_group*, *Parabacteroides*, *Bacteroides*, and *Prevotellaceae_Ga6A1_group.* Notably, PC(22:5(4Z,7Z,10Z, 13Z,16Z)/0:0), PC(22:6-(4Z,7Z,10Z,13Z,16Z,19Z)/22:4(7Z,10Z, 13Z,16Z)), LysoPC(22:6(4Z,7Z,10Z,13Z,16Z,19Z)), and LysoPC(18:1(11Z)) were positively correlated with *Prevotella_9*, *Treponema_2*, *Roseburia*, *Dubosiella*, and *Lactobacillus* and negatively correlated with *Escherichia–Shigella*, *Ruminococcaceae_UCG–014*, others, and *Allobaculum.* Together, these data suggested that some species of gut microbial species may modulate the levels of glycerophospholipid that are correlated with liver regeneration.

## Discussion

The crosstalk between the gut and liver make probiotics play an important role in the progression of liver diseases (Shavandi et al., 2020). Many studies have demonstrated the beneficial effects of probiotics on the modulation of alcohol-induced liver injury, D-galactosamine-induced rat liver injury, and chronic liver disease patients (Nardone et al., 2010). However, to the best of our knowledge, few studies have focused on its effect on liver regeneration. In our research, pretreatment with *L. plantarum* AR113 was found to have increased hepatocyte proliferation and accelerated liver regeneration of PHx rats. The beneficial effects of *L. plantarum* AR113 were associated with increased hepatocyte proliferation, improved liver function, and modulation of gut microbiome and plasma metabolome.

It has been reported that although liver regeneration results ultimately in restoration of liver mass and function, partial hepatectomy is primarily a compensatory hyperplasia (Michalopoulos and Bhushan, 2021). To evaluate the degree of liver injury after 70% partial hepatectomy, liver function was examined. Previous research showed that serum ALT and AST activity increased rapidly and significantly after 70% partial hepatectomy on Day 1 and returned almost to pre-operative levels after 2–3 days in control rats (Yu et al., 2018). Not surprisingly, our study found that *L. plantarum* AR113 administration does not reduce serum ALT and AST activity and ALB II after PHx at Day 3 and Day 7. This is because local inflammatory reactions may occur in response to damage around the ligated area of the liver, resulting in transient increases in serum ALT and AST activity.

Cytokines such as TNF-α, IL-6, HGF, TGF-β, and TNF-α and the activation of NF-κB by cytokines were shown to be required for the initiation of liver regeneration. Several lines of evidence suggest that TNF-α and IL-6 are among the most crucial components of the early signaling pathways leading to regeneration (Fathi et al., 2021). In the liver, IL-6 is secreted by Kupffer cells, and this secretion is stimulated by TNF-α (Jin et al., 2021). HGF is a 97-kd protein that was originally isolated from the peripheral blood of animals after PHx, which is known to be essential to initiate the process of liver regeneration (Gao and Peng, 2021); it could rapidly be elevated by 10- to 20-fold at the early stage of liver injury (Shi and Line, 2020). Present study showed that *L. plantarum* AR113 given two weeks before PHx significantly increased the expression of TNF-α after PHx. but giving probiotics after PHx did not achieve the same effect. In addition, we found that TGF-β was increased in AR113+ PHx group at day 7 after PHx. TGF-β is produced principally in the hepatic stellate cells, and is a representative mitoinhibitory factor, which presumably induces the termination of liver regeneration (Masuda et al., 2020). Our study showed that whether probiotics are given before or after PHx will affect the expression of cytokine expression level.

Accumulating evidence has indicated that the gut microbiome is involved in the pathogenesis of liver diseases by influencing the host’s immunity and metabolism (Lederer et al., 2017). Firmicutes and Bacteroidetes are the two most dominant bacterial phyla affecting host energy extraction efficiency and linked with excess adiposity (Behari et al., 2021). In the present study, *Lactobacillus*, *Lachnospiraceae_NK4A136*, *Ruminococcus_1*, and *[Ruminococcus]_torques* which were affected by *L. plantarum* AR113 administration belong to Firmicutes. *Prevotella_9*, *Bacteroides*, *Alloprevotella*, *Prevotellaceae_Ga6A1_group*, and *Butyricimonas* belong to Bacteroidetes. Some studies have used the ratio of the two dominant phyla (Firmicutes and Bacteroidetes) as a marker for microbial dysbiosis (Jasirwan et al., 2021). Changes in this ratio have also been found in several metabolic disorders. Our study showed that the F/B ratio in the AR113+PHx group was the lowest among all the groups, suggesting that *L. plantarum* AR113 given 2 weeks before PHx could change the microbiome composition. The depletion of genera *Alloprevotella* and *Prevotella* can contribute to non-alcoholic fatty liver disease and microbiome dysbiosis (Safari and Gerard, 2019). In addition, genus *Prevotella* is considered beneficial for promoting hepatic glycogen storage and improving glucose metabolism (Monga Kravetz et al., 2020). We found the expression level of *Prevotella* was decreased after AR113 administration while *Alloprevotella* was increased. Meanwhile, the overgrowth of *Ruminococcus* may lead to metabolic dysfunction and aggravation of liver injury. The genus *Ruminococcus* is considered a gut microbiota signature of non-alcoholic fatty liver disease and was positively correlated with the levels of ALT, AST, TBil-V, and TBA (Demir et al., 2020). Our study found the expression level of *Prevotella* was decreased after AR113 administration. Besides, the colonization of *L. plantarum* AR113 enriched the normal gut microbiota, especially *Lactobacillus*, which can ferment nutrients into lactic acid and benefit health. The decrease in potential pathogens and restoration of the normal gut microbiota by *L. plantarum* AR113 might improve host metabolism and accelerate liver regeneration.

Branched chain amino acids, including Leu, Ile, and Val, play critical roles in regulating metabolism of glucose, lipid, protein synthesis, intestinal health, and immunity (Hernandez-Conde et al., 2021). We found L-leucine and L-isoleucine were up regulated in the *L. plantarum* AR113 administration group compared with the PHx group. Leu was reported to have a proliferative effect on hepatocyte, suggesting that *L. plantarum* AR113 may promote liver regeneration by up-regulating serum Leu. Thus far, the effects of Ile on liver regeneration were limited.

Except for branched chain amino acids, our study also found that *L. plantarum* AR113 administration had an effect on glycerophospholipid metabolism that was evidenced by altered serum levels of glycerophosphoinositol (PIs), PCs, LysoPC, and PEs which are the fundamental components of lipid bilayers of cell membranes. Phospholipids, including PEs, PCs, glycerophosphoserines (PSs), glycerophosphoglycerols (PGs), and CLs, are the prominent membrane lipids (Xie et al., 2016). PIs were ubiquitous components of eukaryotic cells that participate in cell proliferation and survival. It was reported that liver regeneration was characterized by increases in PE, and decreases in glycerophosphoethanolamine (GPE) and glycerophosphocholine (GPC) (Zakian et al., 2005). Indeed, PHx caused a transient and reversible accumulation of phospholipids in the liver at the early phase of liver regeneration. In our study, the probiotic treated group showed greater reductions in PI(20:3(5Z,8Z,11Z)/20:3(8Z,11Z,14Z)), PI(20:4(5Z,8Z,11Z,14Z)/18:0), PC(22:4(7Z,10Z,13Z,16Z)/22:6 (4Z,7Z,10Z,13Z,16Z,19Z)), PC(22:6(4Z,7Z,10Z,13Z,16Z,19Z)/ 22:4(7Z,10Z,13Z,16Z)), 1-Oleoylglycerophosphoserine, and 1-(2-methoxy-6Z-heptadecenyl)-sn-glycero-3-phosphoserine, but had increased concentrations of PE(P-16:0/0:0), sphinganine 1-phosphate, PE(18:1(9Z)/0:0), LysoPE(18:2(9Z,12Z)/0:0), LysoPE(20:4(5Z,8Z,11Z,14Z)/0:0), and LysoPE(0:0/20:4(5Z,8Z,11Z,14Z)). PE is an important lipid marker of inflammation in glycerophospholipid metabolism. An increased concentrations of PE has been detected in systemic circulation of nonalcoholic steatohepatitis patients (Puri et al., 2009). LysoPE has also been shown to play a role in intercellular signaling and in the activation of signaling enzymes (Yanagida and Valentine, 2020), and has been suggested to act through putative G protein-coupled receptors (GPCRs) (Kaluarachchi et al., 2018).

Based on these results, our study found that probiotics intervention can affect the metabolic changes of rats after PHx. More importantly, we also found that *L. plantarum* AR113 administration preoperatively had different effects on liver regeneration rates and metabolites compared with probiotics given postoperatively. Except for the glycerophospholipid metabolism, retrograde endocannabinoid signaling, sphingolipid metabolism, and GPI-anchor biosynthesis pathways, we also found autophagy and ARA metabolism pathways were affected by different ways of probiotics administration. ARA is one of the most abundant polyunsaturated fatty acids present in human tissue and represents one of the pivotal signaling molecules involved in the initiation and propagation of diverse signaling cascades regulating inflammation, pain, and homeostatic function (Turolo et al., 2021). In humans and mammals, ARA has been widely observed to reduce the triacylglycerol (TG) accumulation in liver, plasma, and adipose tissue (Pei et al., 2020). At present, the relationship between ARA and liver regeneration remains unclear. In contrast to ARA metabolism pathways, autophagy was reported to be essential for liver regeneration. Autophagy is a homeostatic mechanism that regulates turnover of long-lived or damaged proteins and organelles and supplies amino acids taken from degradation products of the autolysosome (Xu et al., 2020). To our knowledge, our study first reported that different ways of probiotic administration modulate liver regeneration by affecting autophagy pathways.

Our data showed that LysoPE and PE showed a positive correlation with *Lactobacillus*, *Allobaculum*, *Dubosiella*, *Prevotella*, *Roseburia*, *Butyricimonas*, *Parabacteroides*, and *Bacteroides*, but an inverse correlation with *Helicobacter*, *Ruminococcus*, and *Ruminococcaceae.* The opposite correlation pattern suggests that their counterbalancing role in modulating lipid homeostasis is beneficial for liver regeneration. Thus, the constantly changing gut flora acted as an entire system and exerted various functions on host–microbial nutritional utilization throughout the course of the liver regeneration to meet diverse cell proliferation and energy demands during the different biological processes (Macchi and Sadler, 2020).

## Conclusion

The present study showed that *L. plantarum* AR113 administration increased hepatocyte proliferation and accelerated liver regeneration of PHx rats. Two weeks of *L. plantarum* AR113 before PHx induced decreased F/B ratio. The colonization of *L. plantarum* AR113 enriched the normal gut microbiota, especially *Lactobacillus*. The decrease in potential pathogens and restoration of the normal gut microbiota by *L. plantarum* AR113 might improve host metabolism and accelerated liver regeneration. One of the most profound changes was *L. plantarum* AR113 administration of glycerophospholipid metabolism that was evidenced by decreased serum levels of PI and PCs, and increased LysoPC and PEs. Further investigations will focus on the key metabolites and ultimately clarify the molecular basis for these microbe–host interactions during liver regeneration.

## Data Availability Statement

The original contributions presented in the study are included in the article/Supplementary Material, further inquiries can be directed to the corresponding author/s.

## Ethics Statement

The animal study was reviewed and approved by the Hunan SJA Laboratory Animal Co., Ltd.

## Author Contributions

ChuX and ZhoZ conceptualized the study and wrote and prepared the original draft. MY was in charge of the project administration. CC conceptualized the study. YZ and ZuoZ designed the experiments. WG and ChaX supervised the study. LY and ZH wrote the review. LA and YP conceived and supervised the work. All authors contributed to the article and approved the submitted version.

## Conflict of Interest

The authors declare that the research was conducted in the absence of any commercial or financial relationships that could be construed as a potential conflict of interest.

## Publisher’s Note

All claims expressed in this article are solely those of the authors and do not necessarily represent those of their affiliated organizations, or those of the publisher, the editors and the reviewers. Any product that may be evaluated in this article, or claim that may be made by its manufacturer, is not guaranteed or endorsed by the publisher.

## Footnotes

1
www.Metaboanalyst.ca

2
http://www.genome.jp/kegg/

## Abbreviations

*L. plantarum*: *Lactiplantibacillus plantarum*
PHx: 70% partial hepatectomy
SD: Sprague-Dawley
TNF-α: tumor necrosis factor-α
HGF: hepatocyte growth factor
TGF-β: transforming growth factor-β
PE: phosphatidyl ethanolamine
PC: phosphatidyl choline
PS: phosphatidyl serine
LysoPC: lysophosphatidyl choline
TGF-α: transforming growth factor-α
IL-6: interleukin-6
BAs: bile acids
MRS: Man-Rogosa-Sharpe
PBS: phosphate buffer saline
H&E: hematoxylin-eosin
TBil-V: total bilirubin
ALT: alanine aminotransferase
AST: aspartate aminotransferase
ALB II: albumin II
Glo II: globulin II
TP: total protein
PCoA: principal coordinates analysis
LEfSe: linear discriminant analysis effect size
LDA: linear discriminant analysis
PCA: principle component analysis
OPLS-DA: orthogonal partial least-squares-discriminant analysis
VIP: variable importance in the projection
KEGG: Kyoto Encyclopedia of Genes and Genomes
ANOVA: analysis of variance
Leu: leucine
Ile: isoleucine
Val: valine
PIs: glycerophosphoinositol
PCs: glycerophosphocholines
PSs: glycerophosphoserines
PGs: glycerophosphoglycerols
GPE: glycerophosphoethanolamine
GPC: glycerophosphocholine
GPCRs: G protein-coupled receptors
GPI: glycosylphosphatidylinositol
ARA: arachidonic acid
TG: triacylglycerol.
