# Supplementary material for: Lactiplantibacillus plantarum AR113 Exhibit Accelerated Liver Regeneration by Regulating Gut Microbiota and Plasma Glycerophospholipid
Source: Front Microbiol. 2022 Jan 13;12:800470. doi: 10.3389/fmicb.2021.800470 (PMC8834300; doi:10.3389/fmicb.2021.800470)
Supplement: Supplementary file 1 [file Table_1.docx]

Supplemental table 1 The percentages of Ki-67 positive cells after PHx

|  | | 3d | 7d |
| --- | --- | --- | --- |
| Control | 0 | | 0 |
| Sham | 0 | | 0 |
| PHx | | 0.18±0.06 | 0.19±0.03 |
| AR113+ PHx | | 0.44±0.13 | 0.21±0.06 |
| PHx+ AR113 | | 0.21±0.07 | 0.25±0.06 |
