# Supplementary material for: Lactiplantibacillus plantarum AR113 Exhibit Accelerated Liver Regeneration by Regulating Gut Microbiota and Plasma Glycerophospholipid
Source: Front Microbiol. 2022 Jan 13;12:800470. doi: 10.3389/fmicb.2021.800470 (PMC8834300; doi:10.3389/fmicb.2021.800470)
Supplement: Supplementary file 10 [file Image_1.pdf]

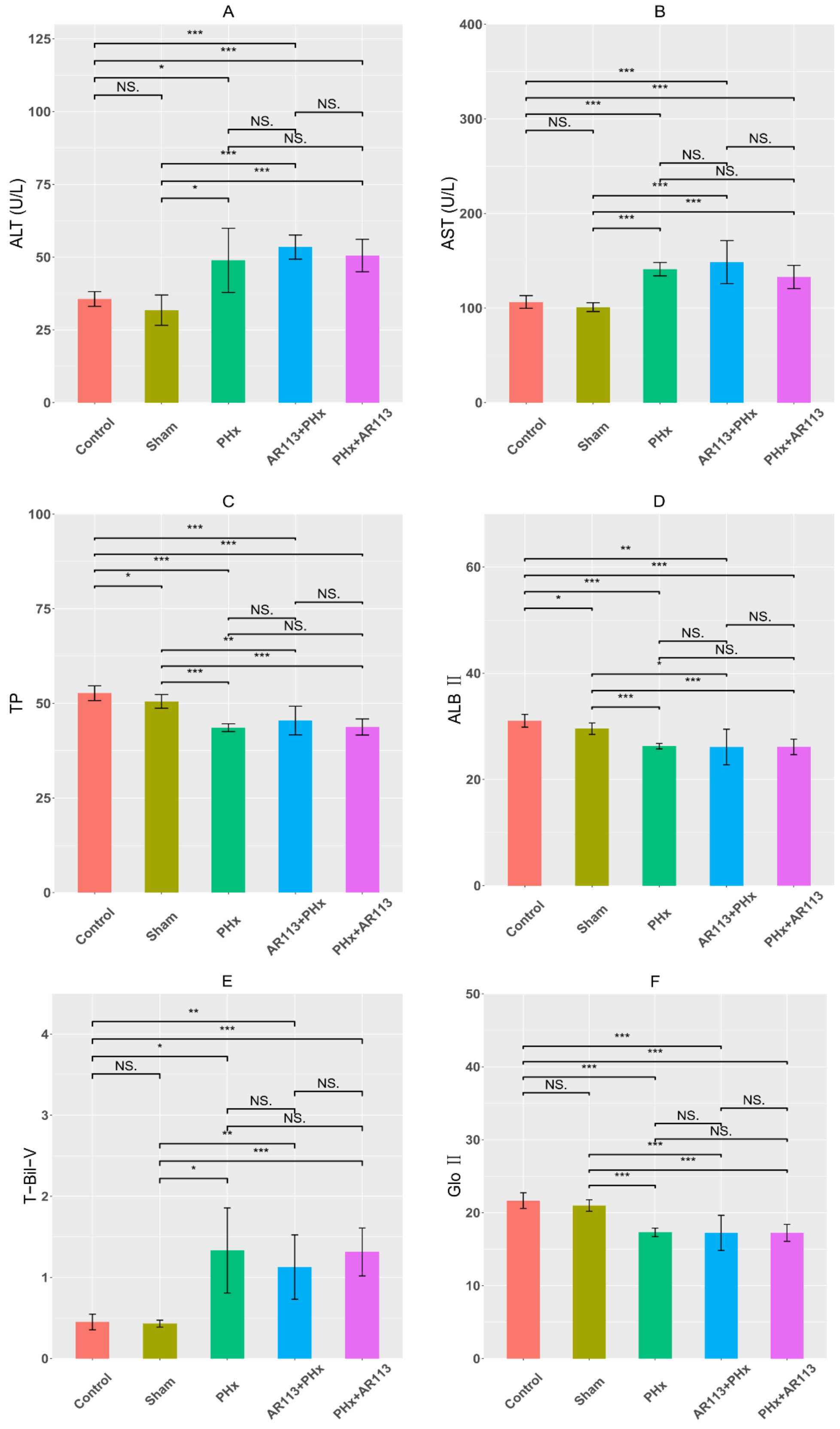

Fig. S1. Liver function at 3 days after PHx among the Control, Sham, PHx, AR113+PHx and PHx+AR113 groups. *$p<0.05$, **$p<0.01$, ***$p<0.001$, ns=not significant as determined by t-test.

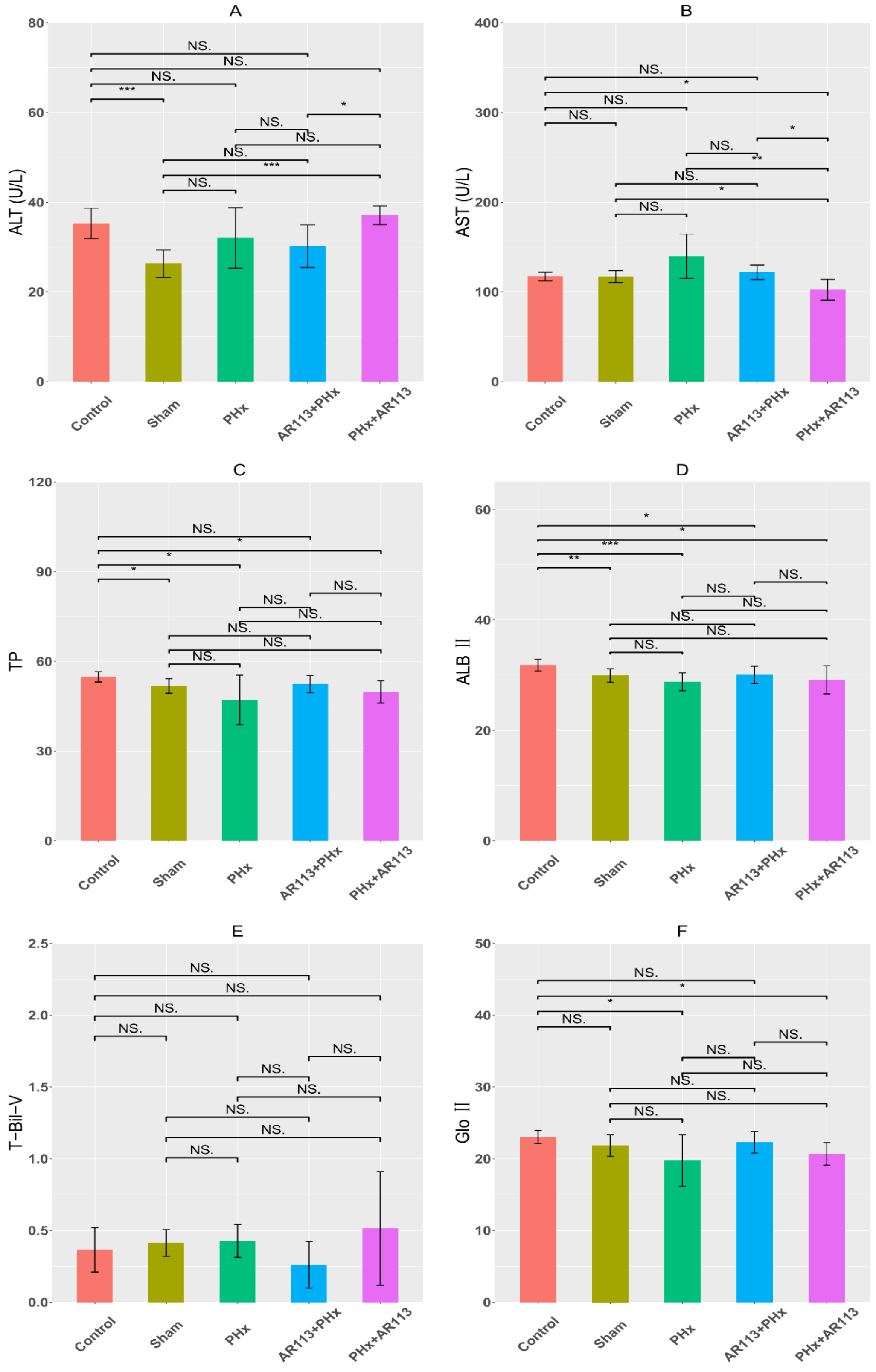

Fig. S2. Liver function at the 7 days after PHx among the Control, Sham, PHx, AR113+PHx and PHx+AR113 groups. *$p<0.05$, **$p<0.01$, ***$p<0.001$, ns=not significant as determined by t-test.

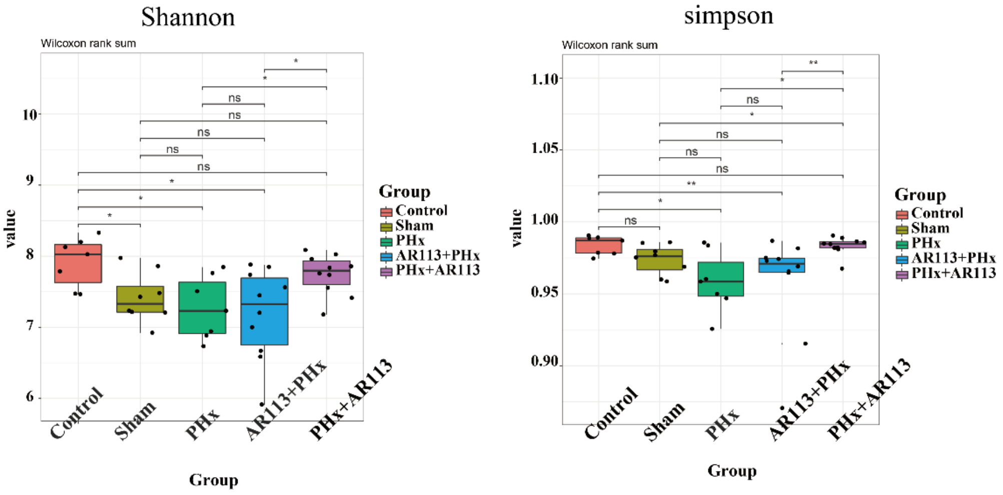

Fig. S3. Comparison of microbial alpha diversity at 3 days after PHx among the control, Sham, PHx, AR113+PHx and PHx+AR113 groups. (A)Shannon index of Diversity; (B) Simpson index of Diversity. *$p<0.05$, **$p<0.01$, ns=not significant as determined by t-test.

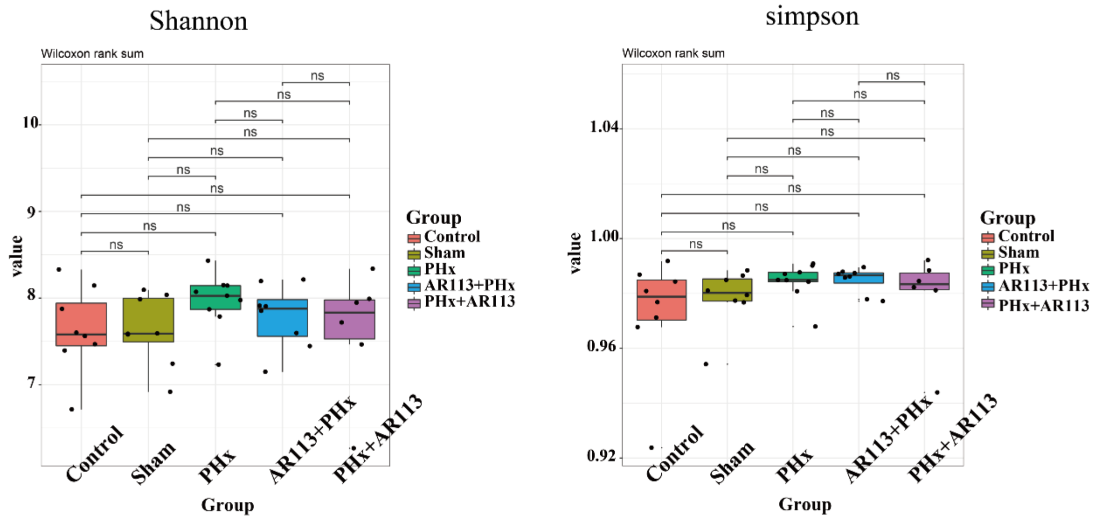

Fig. S4. Comparison of microbial alpha diversity at 7 days after PHx among the control, Sham, PHx, AR113+PHx and PHx+AR113 groups. (A)Shannon index of Diversity; (B) Simpson index of Diversity. ns=not significant as determined by t-test.

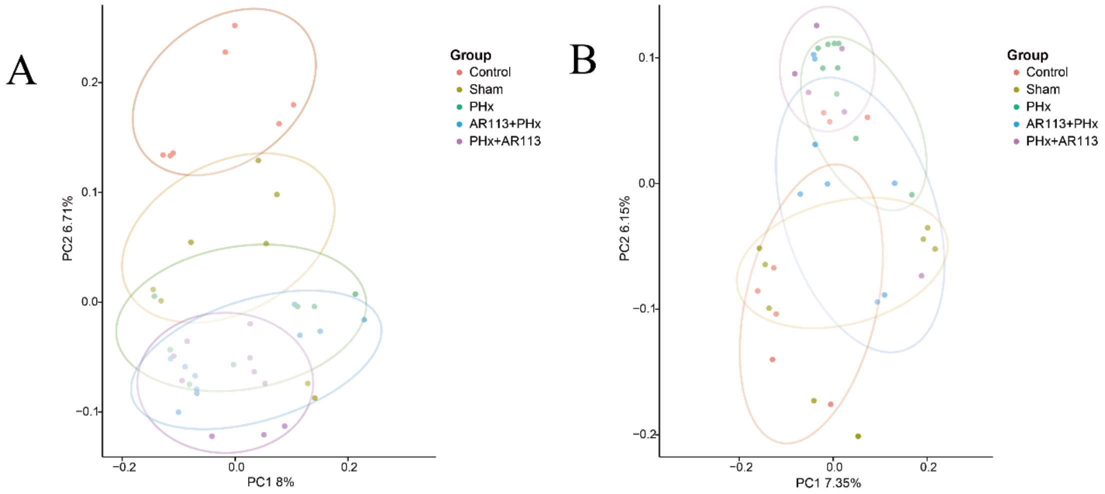

Fig. S5. Principal Coordinates Analysis (PCoA) plot of taxon abundance based on weighted unifrac distance. (A) 3 days after PHx. (B) 7 days after PHx.
